# Chemotherapy is a risk factor of lymphopenia before adjuvant radiotherapy in breast cancer

**DOI:** 10.1002/cnr2.1525

**Published:** 2021-08-14

**Authors:** Fang Chen, Lingyu Ma, Qian Wang, Manling Zhou, Yaqing Nong, Haiman Jing, Ying Han, Yaya Liu, Yulin Hu, Hao Yu, Pingfu Fu, Feng‐Ming ( Spring) Kong

**Affiliations:** ^1^ Department of Clinical Oncology The University of Hong Kong‐Shenzhen Hospital Shenzhen China; ^2^ Biomedical Engineering Shenzhen Polytechnic Shenzhen China; ^3^ Department of Population and Quantitative Health Sciences Case Western Reserve University Cleveland Ohio USA

**Keywords:** breast cancer, chemotherapy, lymphopenia, radiotherapy, risk factors

## Abstract

**Background:**

Lymphopenia can decrease immune function of the host and is a known risk factor for poor prognosis in malignant tumors. Radiation induced lymphopenia was common in patients with breast cancer and was also reported to have a negative effect on long‐term outcome.

**Aims:**

Lymphopenia may be associated with baseline immune status before radiotherapy (RT). This study aimed to explore the rate and risk factors of lymphopenia before start of the adjuvant RT in patients with breast cancer.

**Methods:**

Patients with invasive breast cancer treated from March 2015 to February 2020 and with peripheral lymphocyte counts (PLC) available within 7 days from the beginning of RT were eligible for this study. Data were presented as mean and 95% confidence interval unless otherwise specified. The risk factors of low PLC before RT were identified using univariate and multivariable linear regressions.

**Results:**

A total of 1012 consecutive patients met the study criteria. The mean PLC before RT commencement was 1.58*10^9^/L (95%CI: 1.55–1.62*10^9^/L) with 15.2% (95%CI: 13.1%–17.6%) CTCAE defined lymphopenia, rendering 12.3%, 2.6%, 0.3%, and 0% for grade 1, 2, 3 and 4 respectively. Univariate and multivariable linear regression showed prior chemotherapy was the most significant risk factor (*p* < .001) for low PLC, while age, menopausal status and lymph node stage were not (all *p*s > .05). A total of 912 (90.1%, 95%CI: 88.1%–91.9%) patients had chemotherapy before adjuvant RT in this study. In patients with HR+/HER2‐ breast cancer, 69.0% (95%CI: 63.0%–74.5%) N0 and 98.1% (95%CI: 95.1%–99.5%) N1 had also received chemotherapy.

**Conclusions:**

Patients with breast cancer might have lymphopenia from prior chemotherapy at the start of adjuvant RT which could have negative effect on long‐term outcome. It is also noted that most of the patients with HR+/HER2‐, early‐stage breast cancer were treated with aggressive chemotherapy without knowing the risk of chemotherapy induced lymphopenia. Future study on predictive or prognostic multigene assays is warranted to avoid unnecessary chemotherapy and subsequent lymphopenia in patients with low risk breast cancer.

## 1 INTRODUCTION

The immune escape of malignant cells and lack of immune biomarkers such as lymphocyte are associated with the development, metastasis and recurrence of malignant tumors. One meta‐analysis showed patients with HIV infection and immunodeficiency after transplantation had higher incidence of malignant tumors.^1^ Lymphopenia was found in about 20% of advanced breast cancer, pancreatic cancer, lymphoma and other malignant tumors, while only in 3% of early stage cancers.^2^, ^3^ Meanwhile, various studies had reported a significant association between lymphopenia and poor prognosis in patients with various types of malignant tumors,^4^, ^5^, ^6^, ^7^ including breast cancer.^2^, ^8^, ^9^, ^10^ Indeed, lymphocyte is considered as one of major immunoactive elements which inhibit tumor cell growth.

Lymphocyte also plays an important role for the tumor control effect of immunotherapy including immunocheckpoint inhibitors in malignant tumors and patients with high peripheral lymphocyte count (PLC) had higher immune response rate.^6^ Immunotherapy as an important strategy of precision therapy, has been widely used in melanoma, lung cancer, head and neck cancers, breast cancer and other malignant tumors in recent years. However, only about 20% patients with malignant tumors can benefit from immunotherapy.^11^ Most patients have primary or acquired resistance to immunotherapy. It is believed that lymphopenia and deficiency of functional lymphocyte subsets contribute to the resistance of immunocheckpoint inhibitors. PLC, CD8 + T lymphocyte infiltration, and the expression of immunocheckpoint inhibitors and inhibitor conjugates may be potential markers to predict the efficacy of immunotherapy.^11^ Therefore, in spite of the peripheral white blood cells (WBC), platelets and neutrophils which are related to the toxicity of anti‐tumor therapy, it is also essential to pay attention to PLC before and during the course of treatment. Studying the risk factors of lymphopenia or low PLC has a clinical significance.

Radiation induced lymphopenia was also reported to be a potential risk factor for poor survival in breast cancer^12^, ^13^ and several other malignant tumors.^14^, ^15^, ^16^ RIL was common in patients with breast cancer with approximately 50% patients had lymphopenia after radiotherapy (RT) in Sun's study.^13^ We recently reported that 60.5% patients had lymphopenia after RT, and baseline lymphocyte counts and radiation dosimetric factors to the immune system in radiation field might have contribute to RIL.^17^ This study aimed to preliminarily explore the rate and risk factors of lymphopenia before adjuvant RT in patients with breast cancer, so as to provide a basis for subsequent study of RIL.

## 2 METHODS

### 2.1 Study population

Patients with pathology confirmed invasive breast cancer, aged 18‐year old and above who received adjuvant RT between March 2015 to February 2020 in the University of Hong Kong‐Shenzhen Hospital formed the original study population. Other eligibility criteria included PLC within 7 days from the commencement of RT in this same hospital. Exclusion criteria: stage 0 (DICS), stage IV or recurrent breast cancer, accompanied with immune related diseases.

### 2.2 Data collection

Patient factors (e.g., age, menopausal status), tumor factors (e.g., ER/PR/HER2 subtype, N stage, stage), treatment related parameters (e.g., chemotherapy, anti‐HER2 target therapy and surgical approach) and PLC within 7 days from the beginning of RT were retrospectively collected. Modified N stage and modified stage were applied in this study for patients received neoadjuvant chemotherapy (NACT). Modified (N) stage used the higher (N) stage between clinic (N) stage and pathological (N) stage for patients who had received NACT, and used pathological (N) stage for patients who did not receive NACT. According to Common Terminology Criteria for Adverse Events (CTCAE) version 5.0, lymphopenia was classified using PLC cut‐off of lower limits of normal (LLN)‐0.8*10^9^, 0.8–0.5*10^9^, 0.5–0.2*10^9^ and 0.2*10^9^/L, for grade 1, 2, 3, and 4, respectively. LLN of lymphocyte count was 1.06*10^9^/L in our institution and PLC less than 1.06*10^9^/L was defined as lymphopenia in this study.

### 2.3 Statistical considerations

The primary endpoint of this study was PLC before RT, which was a surrogate endpoint for lymphopenia before RT. The effects of potential risk factors such as age, menopausal status, stage, ER/PR/HER2 subtype, chemotherapy, anti‐HER2 target therapy and surgical approach were estimated using univariate analysis initially and those significant (*p* < .05) in univariate linear analysis were further evaluated by multivariable regression. To avoid the unstable and imprecise estimates of the coefficients, the potential presence of collinearity was assessed using variance inflation factor (VIF) <10 and some of the highly correlated variables with VIF >10 were excluded in the final multivariable regression analysis. Data were presented as mean (95% CI) unless otherwise specified. *p*‐value (*p*) less than .05 was considered to be statistically significant. Statistical analysis was performed using R software (version 3.6.2; https://www.R-project.org).

## 3 RESULTS

### 3.1 Characteristics of patients and prior treatment

**FIGURE 1:**
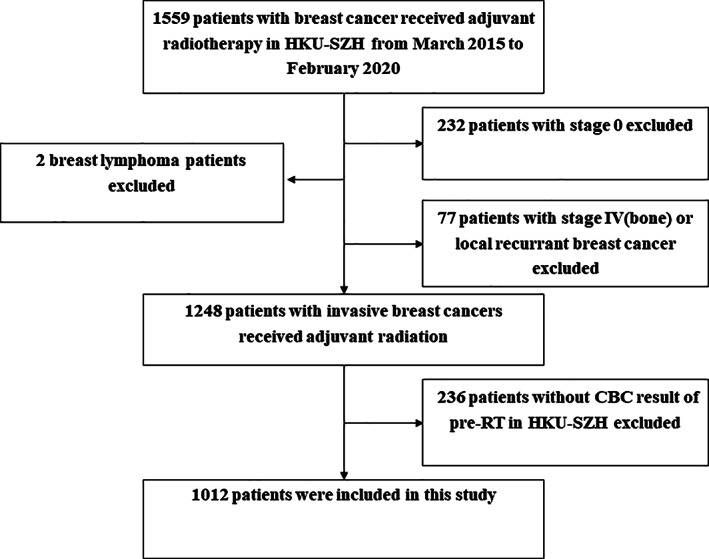
Study population profile. As shown, between March 2015 to February 2020, a total of 1012 patients were included in the study

Between March 2015 to February 2020, a total of 1559 patients with breast cancer received adjuvant RT in the University of Hong Kong‐Shenzhen Hospital. A total of 1012 patients were eligible for this study (Figure 1). The median age was 45 years (range 26–86) with most of the patients being premenopausal (75.3%). The rates of patients with HR‐positive/HER2‐negative (HR+/HER2‐), HER2‐positive (HER2+) and triple‐negative were 60%, 25.6% and 14.4%, respectively. Nine hundred and twelve (90.1%, 95%CI: 88.1%–91.9%) patients received chemotherapy before adjuvant RT (Table 1).

**TABLE 1:** Univariate and multivariable regression analysis of peripheral lymphocyte counts (PLC) before radiotherapy

	Patients	Univariate analysis		Multivariable analysis	
	*n* (%)	Coefficient (95%CI)	*p* value	Coefficient (95%CI)	*p* value
Age					
Median (range) — year	45 (26–86)	0.007 (0.004, 0.011)	<.001	0.003 (−0.002, 0.007)	.270
Menopausal status — no. (%)					
Premenopausal	762 (75.3%)	0		0	
Postmenopausal	250 (24.7%)	0.146 (0.068, 0.223)	<.001	0.036 (−0.019, 0.090)	.199
Modified N stage^a^— no. (%)					
N0	394 (38.9%)	0		0	
N+	618 (61.1%)	−0.075 (−0.143, −0.006)	.033	0.021 (−0.050, 0.091)	.568
Modified N stage^a^— no. (%)					
N0	394 (38.9%)	0			
N1	365 (36.1%)	−0.068 (−0.145, 0.009)	.086		
N2	148 (14.6%)	−0.074 (−0.177, 0.028)	.155		
N3	105 (10.4%)	−0.099 (−0.215, 0.018)	.098		
Modified stage^b^— no. (%)					
I (IA/IB)	261 (25.8%)	0			
II (IIA/IIB)	465 (45.9%)	−0.056 (−0.138, 0.027)	.186		
III (IIIA/IIIB/IIIC)	286 (28.3%)	−0.092 (−0.183, −0.001)	.048		
ER— no. (%)					
Negative	266 (26.3%)	0			
Positive	746 (73.7%)	0.016 (−0.06, 0.092)	.673		
PR— no. (%)					
Negative	335 (33.1%)	0			
Positive	687 (66.9%)	0.011 (−0.06, 0.082)	.758		
HER2— no. (%)					
Negative	761 (75.2%)	0			
Positive	251 (24.8%)	0.037 (−0.041, 0.114)	.354		
Subtype— no. (%)					
HR+/HER2‐	607 (60.0%)	0			
HER2+/HR‐	101 (10.0%)	0.119 (0.001, 0.236)	.049		
HER2+/HR+	158 (15.6%)	−0.001 (−0.094, 0.092)	.979		
HR‐/HER2‐	146 (14.4%)	−0.071 (−0.169, 0.027)	.155		
Surgical approaches (Breast)— no. (%)					
Breast conserving therapy	503 (49.7%)	0			
Mastectomy	509 (50.3%)	−0.026 (−0.093, 0.041)	.447		
Surgical approaches (Axillary)— no. (%)					
Sentinel lymph node biopsy only	376 (37.2%)	0			
Axillary lymph node dissection	636 (62.8%)	−0.061 (−0.130, 0.008)	.085		
Margin— no. (%)					
Clear	966 (95.5%)	0			
Close or positive	46 (4.5%)	0.012 (−0.148, 0.173)	.882		
Chemotherapy— no. (%)					
None	100 (9.9%)	0		0	
Yes	912 (90.1%)	−0.485 (−0.593, −0.377)	<.001	−0.474 (−0.590, −0.357)	<.001
Chemotherapy strategy— no. (%)					
None	100 (9.9%)	0			
Neoadjuvant	165 (16.3%)	−0.383 (−0.511, −0.255)	<.001		
Adjuvant	718 (70.9%)	−0.514 (−0.623, −0.405)	<.001		
Neoadjuvant+adjuvant	29 (2.9%)	−0.325 (−0.586, −0.064)	.015		
Chemotherapy regimens— no. (%)					
Anthracycline+taxane combined	641 (63.3%)	0			
Anthracycline+ cyclophosphamide	27 (2.7%)	−0.236 (−0.436, −0.035)	.022		
Taxane+ cyclophosphamide/carboplatin	244 (24.1%)	0.078 (0.001, 0.155)	.046		
None	100 (9.9%)	0.499 (0.389, 0.609)	<.001		
Time interval between last chemotherapy and the start of RT— mean (95%CI) (days)	42.1 (40.2, 44.0)	0.003 (0.002, 0.004)	<.001		
Anti‐HER2 target therapy— no. (%)					
None	771 (76.2%)	0			
Yes	241 (23.8%)	0.044 (−0.034, 0.123)	.270		
Endocrine therapy— no. (%)					
None	243 (24.0%)	0			
Yes	769 (76.0%)	0.035 (−0.043, 0.113)	.381		

^a^ Modified N stage: The higher N stage between clinic N stage and yp N stage for patients who had received neoadjuvant chemotherapy.

^b^ Modified stage: The higher stage between clinic stage and yp stage for patients who had received neoadjuvant chemotherapy.

### 3.2 Lymphopenia before adjuvant radiotherapy

The mean PLC before adjuvant RT was 1.58*10^9^/L (95%CI: 1.55–1.62*10^9^/L) and mean WBC was 5.70*10^9^/L (95%CI: 5.54–5.85*10^9^/L) in this study. One hundred and fifty‐four patients (15.2%, 95%CI: 13.1%–17.6%) patients had lymphopenia, rendering 12.3%, 2.6%, 0.3%, and 0% for grade 1, 2, 3, and 4, respectively. Among the 154 patients with lymphopenia, 16.9% (26/154) patients were triple‐negative, 64.3% (99/154) were HR+/HER2‐ and 18.8% (29/154) were HER2+ subtype.

The mean time from last chemotherapy to the initiation of RT was 42 days (95%CI: 40, 44) in the 912/1012 (90.1%, 95%CI: 88.1%–91.9%) patients who had received chemotherapy and 32 days (95%CI: 31, 34) in the 747/1012 (73.8%, 95%CI: 71.0%–76.5%) patients who had received adjuvant chemotherapy. The mean time from surgery to the initiation of RT was 161 days (95%CI: 157, 164) in 1012 patients and 20 days (95%CI: 18, 23) in the 100/1012 (9.9%, 95%CI: 8.1%–11.9%) patients who had not received chemotherapy.

### 3.3 Univariate and multivariable linear regressions of risk factors for peripheral lymphocyte counts (PLC) before RT

As shown on Table 1, under univariate linear regression, prior chemotherapy, the usage of taxanes and anthracyclines regimen, chemotherapy strategy, time interval between last chemotherapy and the start of RT, age, menopausal status and modified N stage were significantly associated with PLC before RT (*p*s < .05), while ER status, PR status, HER‐2 status, subtype, surgical approaches, endocrine therapy and anti HER‐2 target therapy were not. To avoid the unstable and imprecise estimates of the coefficients, some of the highly correlated variables such as chemotherapy regimens, chemotherapy strategy, time interval between last chemotherapy and the start of RT and modified stage were excluded in the final multivariable regression. Multivariable linear regression showed chemotherapy was the significant risk factor for low PLC before RT (*p* < .001) controlling the effects of age, menopausal status and modified N stage.

### 3.4 Detailed application of chemotherapy before adjuvant RT in breast cancer

The 1012 patients with breast cancer in this study had surgery and systemic therapy in several hospitals in a southern city of China and received RT in our institution. A total of 90.1% (95%CI: 88.1%–91.9%) patients received chemotherapy before adjuvant RT: 70.9% (95%CI: 68.0%–73.7%), 16.3% (95%CI: 14.1%–18.7%), and 2.9% (95%CI: 1.9%–4.1%) patients had adjuvant chemotherapy, neoadjuvant chemotherapy and neoadjuvant combined with adjuvant chemotherapy, respectively. Most patients (61.8%, 95%CI: 58.8%–64.9%) were treated with combination chemotherapy with taxanes and anthracycline (Table 1).

**FIGURE 2:**
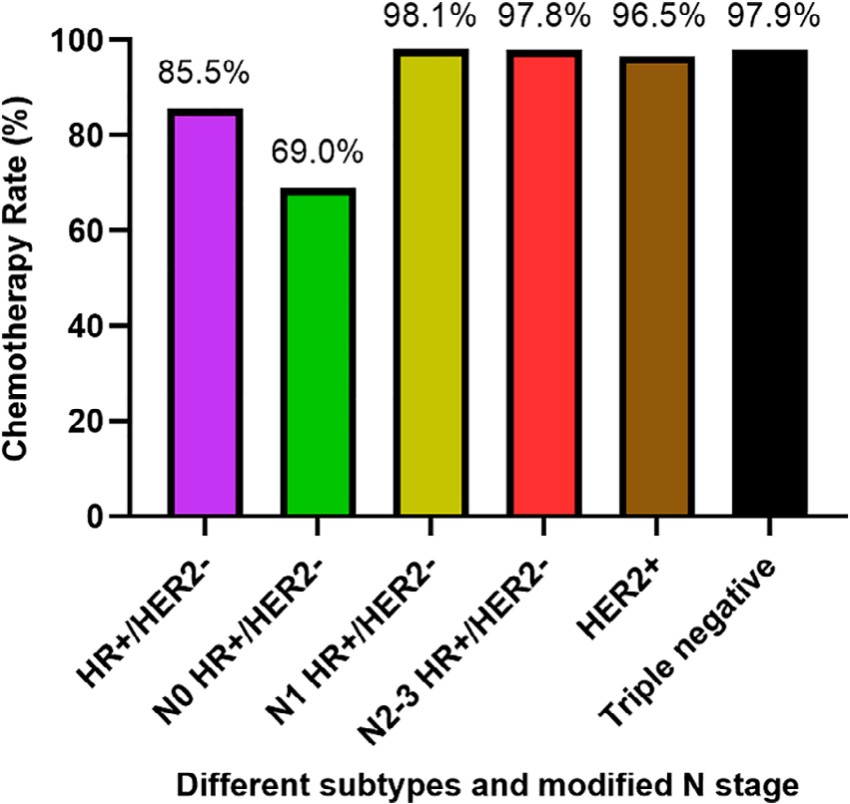
Chemotherapy rate in different subtypes and modified N stage. 69.0% (95%CI: 63.0%–74.5%) modified N0 and 98.1% (95%CI: 95.1%–99.5%) N1 patients with HR+/HER2‐ breast cancer had received chemotherapy. The rate of chemotherapy was 97.8% (95%CI: 93.8%–99.6%), 96.5% (95%CI: 93.5%–98.4%), 97.9% (95%CI: 94.1%–99.6%) in patients with N2‐3 HR+/HER2‐, HER2+, triple negative breast cancer respectively

As shown on Figure 2, vast majority of patients with aggressive breast cancer had chemotherapy, as expected. In 259 patients with HER2‐positive and 146 patients with triple negative subtypes, the rates of chemotherapy were 96.5% (95%CI: 93.5%–98.4%) and 97.9% (95%CI: 94.1%–99.6%), respectively. In 139 patients with HR+/HER2‐ locally advanced (N2‐3) breast cancer, 97.8% (95%CI: 93.8%–99.6%) patients received chemotherapy. In 607 patients with HR+/HER2‐, 62.8% (95%CI: 55.6%–69.7%) patients with stage I had chemotherapy. In addition, 69.0% (180/261, 95%CI: 63.0%–74.5%) modified N0 and 98.1% (203/207, 95%CI: 95.1%–99.5%) modified N1 patients with HR+/HER2‐, early‐stage breast cancer received chemotherapy (Figure 2). Chemotherapy was a significant factor for lymphopenia in this subgroup as well (*p* < .001).

**FIGURE 3:**
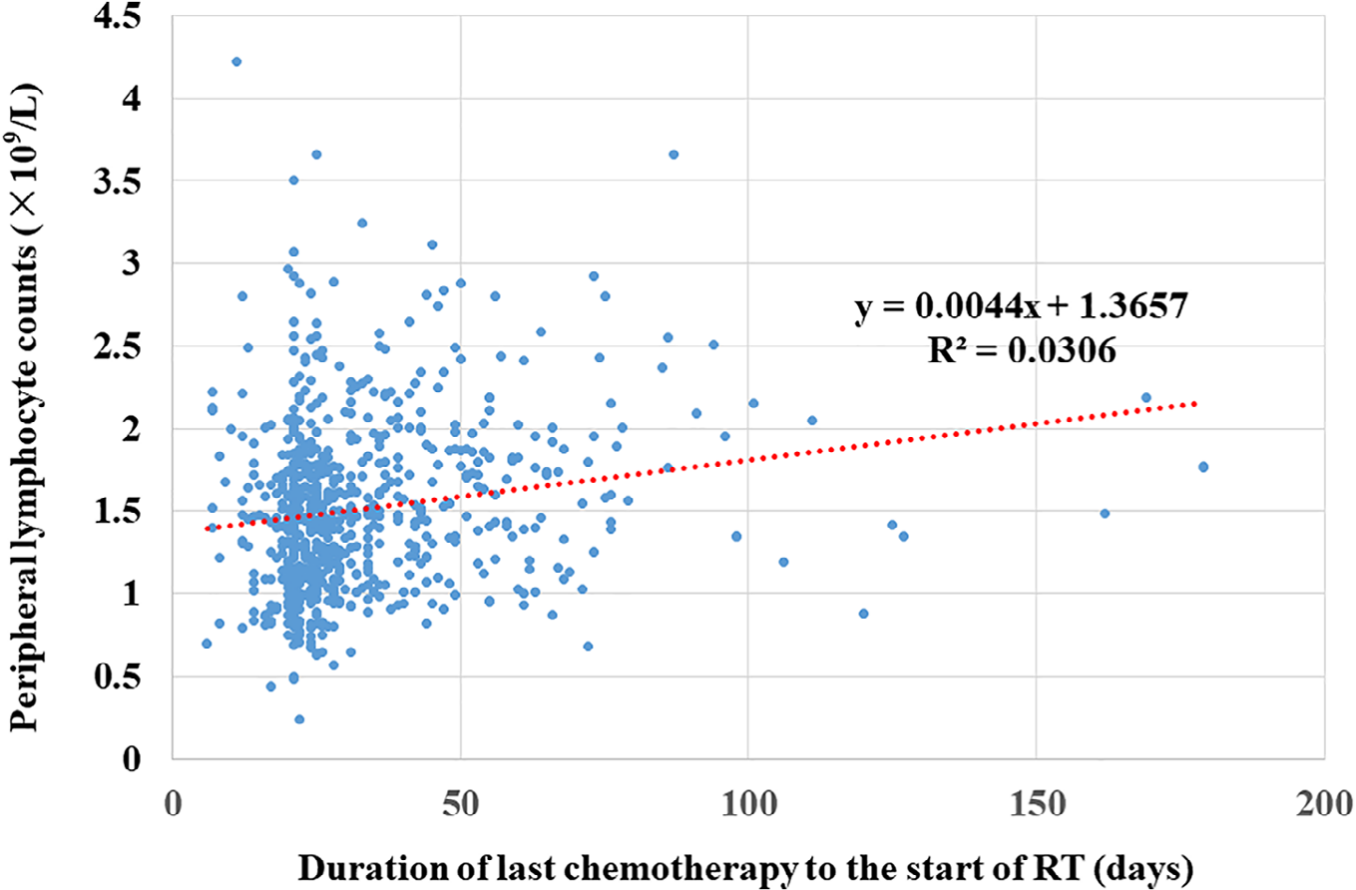
The influence of time interval between last chemotherapy and the start of radiotherapy (RT) on peripheral lymphocyte counts (PLC) before RT. There appeared to have a positive linear relationship between time interval of last chemotherapy to the start of RT and PLC in the 747 patients who had received adjuvant chemotherapy (R^2^ = 0.0306)

Time interval between last chemotherapy and the start of RT was a significant risk factor for low PLC in all patients treated with various regimens of chemotherapy in univariate linear regression (Table 1). As Figure 3 shown, there appeared to have a positive linear relationship between time interval of last chemotherapy to the start of RT and PLC in the 747 patients who had received adjuvant chemotherapy.

## 4 DISCUSSION

This study of 1012 patients demonstrated that 15.2% patients with breast cancer had lymphopenia before the commencement of adjuvant RT, at the time when the bone marrow suppression of previous chemotherapy and inflammatory response of surgery were considered to have recovered according to traditional assessment of the neutrophils and platelets. This study showed prior chemotherapy was the significant risk factor for low PLC before RT, which was consistent with previous report of Sage EK's study.^18^ The benefit of chemotherapy in aggressive type of breast cancer is known, thus the vast majority of these patients had received chemotherapy before adjuvant RT. As unexpected, most patients with HR+/HER2‐, early‐stage breast cancer were also treated with aggressive chemotherapy in this study while chemotherapy is considered to have low clinical benefits in this population.

The most common chemotherapy regimens for breast cancer are anthracycline, taxane and cyclophosphamide. Ménétrier‐Caux et al had reported cyclophosphamide, cisplatin, methotrexate and taxane were the strongest chemotherapeutic agents that induced lymphocyte deficiency.^11^ Kotsakis et al also found taxane can induce lymphocytosis in solid tumors.^19^ Among the 912 patients who had received chemotherapy in this study, 97% patients had received taxane combined chemotherapy. In the meanwhile, 73.2% patients had received anthracycline combined chemotherapy which was also reported to cause lymphocytosis in patients with breast cancer.^20^ Since chemotherapy can induce lymphopenia which was associated with poor long‐term survival in patients with breast cancer^2^, ^8^, ^9^, ^10^ and cause other toxicity (e.g., bone marrow suppression, gastrointestinal adverse events), we should avoid unnecessary aggressive chemotherapy in low risk patients with extremely low clinical benefits and start more effective adjuvant endocrine therapy and radiotherapy without delay.

However, most of our patients with HR+/HER2‐, early‐stage breast cancer (69.0% N0 and 98.1% N1) had received chemotherapy in this study, which may due to the inadequate application of Oncotype Dx, EndoPredict, Mammaprint and other gene expression assays in real‐world practice of southern China. Physicians tended to routinely give aggressive chemotherapy while the evidence of confirmed low risk by multigene assays was absent. 69.0% patients with HR+/HER2‐, node‐negative breast cancer were treated with chemotherapy in this study while only 13.5% patients of this group were high risk who were in true need of chemotherapy by Oncotype DX.^21^ Oncotype DX is the preferred validated multigene assays to predict the benefits of chemotherapy in HR+/HER2‐, node‐negative breast cancer,^22^, ^23^ as well as in HR+/HER2‐ limited node‐positive (N1) breast cancer.^22^, ^24^, ^25^ Since Oncotype DX is not available in China, 70‐gene assay of Mammaprint is another category 1 option to identify the low risk patients who can omit chemotherapy without a detrimental effect.^25^ In OPTIMA prelim trial which compared multiparameter tests in patients with ER+/HER2‐, early‐stage breast cancer, the rate of low‐risk categorized by Mammaprint and Oncotype DX was 61.4% and 54.2%, respectively.^26^ In MINADCT study (21% N1 and 79% node negative respectively), 49% patients of HR+/HER2‐ breast cancer were low risk by both of clinical and genomic results, and additionally 46% patients with high clinic risk were low risk by Mammaprint who could omit chemotherapy at the very beginning.^27^ Therefore, predictive or prognostic multigene assays should be more applied in clinic practice in patients with HR+/HER2‐, early‐stage breast cancer. With the affirmed low risk by quantized genomic results, physicians could be more confident to omit unnecessary chemotherapy in these cases as NCCN recommended^25^ and the risk of lymphopenia in this low risk population might be reduced.

As for high risk patients with locally advanced breast cancer or aggressive subtypes such as triple negative breast cancer (TNBC), it is difficult to omit chemotherapy with the irreplaceable cytotoxic effect and survival benefits in these cases in spite of the possibility of chemotherapy induced lymphopenia.^28^, ^29^ TNBC is considered to be immunogenic with higher PD‐L1 mRNA expression^30^ and CD8+ T cell infiltration.^31^ Keynote522 study demonstrated that neoadjuvant immunocombined chemotherapy could improve the pathological complete response rate of locally advanced TNBC.^32^ The direct tumor cytotoxicity of chemotherapy was reported to increase immunogenic of dead cancer cells and inhibit over‐activation of immunosuppression T cells (e.g., Treg cells).^11^ However, it is not clear whether the effect of immunocombined chemotherapy be weakened by synchronous lymphopenia induced by chemotherapy since patients with lower PLC had lower immune response rate.^6^ Further study is needed to explore the influence of treatment related lymphopenia in patients with TNBC receiving immunocombined chemotherapy. Another important question is timing of RT start, what is the best interval. From PLC point of view, PLC before RT was higher as the time interval between last chemotherapy and the start of RT prolonged. In clinic practice, physicians used to perform RT as the neutropenia from prior chemotherapy recovers. Should PLC and lymphopenia be included in our consideration as well? Further study is needed to investigate the proper timing of RT start in patients with breast cancer receiving adjuvant chemotherapy and subsequent RT.

This study has some limitations: (1) This is an exploring study of lymphopenia before RT without detailed investigation on lymphocyte subsets and immune biomarkers; (2) This is a retrospective study which carries the flaws of such studies; (3) Most patients had received combined chemotherapy with anthracycline and taxane and it was difficult to test the effects of each specific chemotherapy drug on lymphopenia. Further studies are needed to explore the change of detailed immune biomarkers by chemotherapy before RT and the subsequent impact on radiation induced lymphopenia in patients with breast cancer.

## 5 CONCLUSIONS

This study of 1012 patients with breast cancer demonstrated that patients might have lymphopenia before RT which was associated with prior chemotherapy. Since lymphopenia has a negative effect on long‐term survival, further study on predictive or prognostic multigene assays for risk stratification and personalized decision of chemotherapy may help avoid unnecessary chemotherapy and reduce chemotherapy induced lymphopenia in patients with low risk breast cancer.

## CONFLICT OF INTEREST

The authors declare no potential conflict of interest.

## AUTHOR CONTRIBUTIONS

*Primary investigator, Conceptualization, investigation, validation, Resources, Formal Analysis, Methodology, Data Curation, Writing ‐ Original Draft and Final manuscript approval*, F.C.; *Formal Analysis, Methodology and Final manuscript approval*, H.Y., L.M. and P.F.; *Data Curation, Validation and Final manuscript approval*, Q.W., M.Z., Y.N., H.J., Y.H., Y.L. and Y.H.; *Conceptualization, Methodology and Final manuscript approval*, F.‐M. (S.) K.

## ETHICS STATEMENT

The study was conducted in accordance with the Declaration of Helsinki (as revised in 2013). The study was approved by the ethics committee of the University of Hong Kong‐Shenzhen Hospital # 2019 098 and individual consent for this retrospective analysis was waived.

## Data Availability

The datasets analyzed during the current study are not publicly available due to privacy but are available from the corresponding author on reasonable request.

## References

[1] Grulich AE , van Leeuwen MT , Falster MO , Vajdic CM . Incidence of cancers in people with HIV/AIDS compared with immunosuppressed transplant recipients: a meta‐analysis. Lancet. 2007;370(9581):59‐67. 17617273 10.1016/S0140-6736(07)61050-2

[2] Trédan O , Manuel M , Clapisson G , et al. Patients with metastatic breast cancer leading to CD4^+^ T cell lymphopaenia have poor outcome. Eur J Cancer. 2013;49(7):1673‐1682. 23265706 10.1016/j.ejca.2012.11.028

[3] Borg C , Ray‐Coquard I , Philip I , et al. CD4 lymphopenia as a risk factor for febrile neutropenia and early death after cytotoxic chemotherapy in adult patients with cancer. Cancer: Interdiscip Int J Am Cancer Soc. 2004;101(11):2675‐2680. 10.1002/cncr.20688 15503313

[4] Saroha S , Uzzo RG , Plimack ER , Ruth K , al‐Saleem T . Lymphopenia is an independent predictor of inferior outcome in clear cell renal carcinoma. J Urol. 2013;189(2):454‐461. 23041457 10.1016/j.juro.2012.09.166 PMC3545104

[5] Ceze N , Thibault G , Goujon G , et al. Pre‐treatment lymphopenia as a prognostic biomarker in colorectal cancer patients receiving chemotherapy. Cancer Chemother Pharmacol. 2011;68(5):1305‐1313. 21448592 10.1007/s00280-011-1610-3

[6] Ray‐Coquard I , Dussart S , Goillot C , et al. A risk model for severe anemia to select cancer patients for primary prophylaxis with epoetin α: a prospective randomized controlled trial of the ELYPSE study group. Ann Oncol. 2009;20(6):1105‐1112. 19174452 10.1093/annonc/mdn750

[7] Hasenclever D , Diehl V , Armitage JO , et al. A prognostic score for advanced Hodgkin's disease. N Engl J Med. 1998;339(21):1506‐1514. 9819449 10.1056/NEJM199811193392104

[8] De Giorgi U , Mego M , Scarpi E , et al. Relationship between lymphocytopenia and circulating tumor cells as prognostic factors for overall survival in metastatic breast cancer. Clin Breast Cancer. 2012;12(4):264‐269. 22591634 10.1016/j.clbc.2012.04.004

[9] Manuel M , Tredan O , Bachelot T , et al. Lymphopenia combined with low TCR diversity (divpenia) predicts poor overall survival in metastatic breast cancer patients. Oncoimmunology. 2012;1(4):432‐440. 22754761 10.4161/onci.19545 PMC3382902

[10] Afghahi A , Purington N , Han SS , et al. Higher absolute lymphocyte counts predict lower mortality from early‐stage triple‐negative breast cancer. Clin Cancer Res. 2018;24(12):2851‐2858. 29581131 10.1158/1078-0432.CCR-17-1323 PMC6366842

[11] Ménétrier‐Caux C , Ray‐Coquard I , Blay J‐Y , Caux C . Lymphopenia in cancer patients and its effects on response to immunotherapy: an opportunity for combination with cytokines? J Immunother Cancer. 2019;7(1):1‐15. 30922400 10.1186/s40425-019-0549-5 PMC6437964

[12] Cho O , Chun M , Kim SW , Jung YS , Yim H . Lymphopenia as a potential predictor of ipsilateral breast tumor recurrence in early breast cancer. Anticancer Res. 2019;39(8):4467‐4474. 31366546 10.21873/anticanres.13620

[13] Sun G‐Y , Wang S‐L , Song Y‐W , et al. Radiation‐induced lymphopenia predicts poorer prognosis in patients with breast cancer: a post‐hoc analysis of a randomized controlled trial of postmastectomy hypofractionated radiotherapy. Int J Radiat Oncol Biol Phys. 2020;108(1):277‐285. 32147519 10.1016/j.ijrobp.2020.02.633

[14] So TH , Chan SK , Chan WL , et al. Lymphopenia and radiation dose to circulating lymphocyte with neoadjuvant chemoradiation in esophageal squamous cell carcinoma. Adv Radiat Oncol. 2020;5(5):880‐888. 33089018 10.1016/j.adro.2020.03.021 PMC7560564

[15] Wild AT , Ye X , Ellsworth SG , et al. The association between chemoradiation‐related lymphopenia and clinical outcomes in patients with locally advanced pancreatic adenocarcinoma. Am J Clin Oncol. 2015;38(3):259‐265. 23648440 10.1097/COC.0b013e3182940ff9 PMC3991773

[16] Tang C , Liao Z , Gomez D , et al. Lymphopenia association with gross tumor volume and lung V5 and its effects on non‐small cell lung cancer patient outcomes. Int J Radiat Oncol Biol Phys. 2014;89(5):1084‐1091. 25035212 10.1016/j.ijrobp.2014.04.025

[17] Chen F , Jin JY , Wang W , et al. Radiation induced lymphopenia is associated with the effective dose to the circulating immune cells (EDIC) for breast cancer. Int J Radiat Oncol Biol Phys. 2020;108(3):e57‐e58.

[18] Sage EK , Schmid TE , Sedelmayr M , et al. Comparative analysis of the effects of radiotherapy versus radiotherapy after adjuvant chemotherapy on the composition of lymphocyte subpopulations in breast cancer patients. Radiother Oncol. 2016;118(1):176‐180. 26683801 10.1016/j.radonc.2015.11.016

[19] Kotsakis A , Sarra E , Peraki M , et al. Docetaxel‐induced lymphopenia in patients with solid tumors: a prospective phenotypic analysis. Cancer: Interdiscip Int J Am Cancer Soc. 2000;89(6):1380‐1386. 10.1002/1097-0142(20000915)89:6<1380::aid-cncr23>3.0.co;2-r 11002234

[20] Wijayahadi N , Haron MR , Stanslas J , Yusuf Z . Changes in cellular immunity during chemotherapy for primary breast cancer with anthracycline regimens. J Chemother. 2007;19(6):716‐723. 18230556 10.1179/joc.2007.19.6.716

[21] Sparano JA , Gray RJ , Makower DF , et al. Adjuvant chemotherapy guided by a 21‐gene expression assay in breast cancer. N Engl J Med. 2018;379(2):111‐121. 29860917 10.1056/NEJMoa1804710 PMC6172658

[22] Albain KS , Barlow WE , Shak S , et al. Prognostic and predictive value of the 21‐gene recurrence score assay in postmenopausal women with node‐positive, oestrogen‐receptor‐positive breast cancer on chemotherapy: a retrospective analysis of a randomised trial. Lancet Oncol. 2010;11(1):55‐65. 20005174 10.1016/S1470-2045(09)70314-6 PMC3058239

[23] Paik S , Tang G , Shak S , et al. Gene expression and benefit of chemotherapy in women with node‐negative, estrogen receptor‐positive breast cancer. J Clin Oncol. 2006;24(23):3726‐3734. 16720680 10.1200/JCO.2005.04.7985

[24] Gluz O , Nitz UA , Christgen M , et al. West German Study Group Phase III PlanB Trial: first prospective outcome data for the 21‐gene recurrence score assay and concordance of prognostic markers by central and local pathology assessment. J Clin Oncol. 2016;34(20):2341‐2349. 26926676 10.1200/JCO.2015.63.5383

[25] Gradishar WJ , Moran MS , Abraham J , et al. NCCN guidelines® insights: breast cancer, version 4.2021: featured updates to the NCCN guidelines. J Natl Compr Canc Netw. 2021;19(5):484‐493. 34794122 10.6004/jnccn.2021.0023

[26] Bartlett J , Bayani J , Marshall A , et al. Comparing breast cancer multiparameter tests in the OPTIMA prelim trial: no test is more equal than the others. J Natl Cancer Inst. 2016;108(9):djw050. 27130929 10.1093/jnci/djw050 PMC5939629

[27] Cardoso F , van't Veer LJ , Bogaerts J , et al. 70‐gene signature as an aid to treatment decisions in early‐stage breast cancer. N Engl J Med. 2016;375(8):717‐729. 27557300 10.1056/NEJMoa1602253

[28] Sparano JA , Wang M , Martino S , et al. Weekly paclitaxel in the adjuvant treatment of breast cancer. N Engl J Med. 2008;358(16):1663‐1671. 18420499 10.1056/NEJMoa0707056 PMC2743943

[29] Citron ML , Berry DA , Cirrincione C , et al. Randomized trial of dose‐dense versus conventionally scheduled and sequential versus concurrent combination chemotherapy as postoperative adjuvant treatment of node‐positive primary breast cancer: first report of Intergroup Trial C9741/Cancer and Leukemia Group B Trial 9741. J Clin Oncol. 2003;21(8):1431‐1439. 12668651 10.1200/JCO.2003.09.081

[30] Mittendorf EA , Philips AV , Meric‐Bernstam F , et al. PD‐L1 expression in triple‐negative breast cancer. Cancer Immunol Res. 2014;2(4):361‐370. 24764583 10.1158/2326-6066.CIR-13-0127 PMC4000553

[31] Liu S , Lachapelle J , Leung S , Gao D , Foulkes WD , Nielsen TO . CD8+ lymphocyte infiltration is an independent favorable prognostic indicator in basal‐like breast cancer. Breast Cancer Res. 2012;14(2):1‐14. 10.1186/bcr3148 PMC3446382 22420471

[32] Schmid P , Cortes J , Pusztai L , et al. Pembrolizumab for early triple‐negative breast cancer. N Engl J Med. 2020;382(9):810‐821. 32101663 10.1056/NEJMoa1910549

